# A Treatise on Epidemic Cholera and Allied Diseases

**Published:** 1885-09

**Authors:** 


A Treatise on Epidemic Cholera and Allied Diseases. By A. B. PALMER, M. D., LL. D., Professor of Pathology, Practice of Medicines, and Clinical Medicine, University of Michigan. Ann Arbor, Mich. : Register Publishing House. 1885.

We welcome the present work from the pen of Prof. Palmer. It is the product of large experience and study. The author adds a chapter, in which he discusses choleroid disease, including summer diarrhœas, cholera morbus and cholera infantum.

At a period when the profession are anticipating the approach of this disease, the publication of this work comes most opportune, and will be read with interest. We commend the work, and have also a personal interest in it from long acquaintance with the accomplished author.

